# Developing a Suitable Model for Supplier Selection Based on Supply Chain Risks: An Empirical Study from Iranian Pharmaceutical Companies

**Published:** 2012

**Authors:** Gholamhossein Mehralian, Ali Rajabzadeh Gatari, Mohadese Morakabati, Hossein Vatanpour

**Affiliations:** a *Department of Pharmacoeconomics and Pharmaceutical Management, School of Pharmacy, Shaheed Beheshti University of Medical Sciences, Tehran, Iran.*; b *Department of Operation Management, Tarbiat Modares University, Tehran, Iran.*; c *University of Science and Culture, Tehran, Iran.*; d *Pharmaceutical Sciences Research Center, Shaheed Beheshti University of Medical Sciences, Tehran, Iran.*; e *Student Research committee, School of Pharmacy, Shahid Beheshti University of Medical Sciences, Tehran, Iran.*

**Keywords:** Supply chain management, Risk management, Supplier selection, Pharmaceutical industry, Iran

## Abstract

The supply chain represents the critical link between the development of new product and the market in pharmaceutical industry. Over the years, improvements made in supply chain operations have focused largely on ways to reduce cost and gain efficiencies in scale. In addition, powerful regulatory and market forces have provided new incentives for pharmaceutical firms to basically rethink the way they produce and distribute products, and also to re-imagine the role of the supply chain in driving strategic growth, brand differentiation and economic value in the health continuum. The purpose of this paper is to formulate basic factors involved in risk analysis of pharmaceutical industry, and also determine the effective factors involved in suppliers selection and their priorities. This paper is based on the results of literature review, experts’ opinion acquisition, statistical analysis and also using MADM models on data gathered from distributed questionnaires. The model consists of the following steps and components: first factors involved in to supply chain risks are determined. Based on them a framework is considered. According the result of statistical analysis and MADM models the risk factors are formulated. The paper determines the main components and influenceial factors involving in the supply chain risks. Results showed that delivery risk can make an important contribution to mitigate the risk of pharmaceutical industry.

## Introduction

The pharmaceutical industry is defined as a system of processes, operations and organizations involved in the discovery, development and production of drugs and medications. The pharmaceutical supply chain (PSC) signifies the route through which essential pharmaceutical products are distributed to the final end-users at the right quality, at the right place and at the right time (1).

A scientific and technological transformation is occurring in the pharmaceutical industry that will make it possible for drug producers to produce profitable new medicines for situations that cannot be treated very well today and for conditions which have formerly persisted against all treatments. But transformations require adjustments, and this revolution needs the supply chains to be regulated with it (2). The pharmaceutical companies which have long been regarded as the laggards in supply chain management (SCM) have an option: either they can get rid of the short-term pressures they encounter, or they can have a long perspective and recognize the real contribution the supply chains can bring about. In the past, pharmaceutical companies did not consider supply chain management concepts (3). However, now several factors are pushing pharmaceutical companies to modify their conventional approaches of conducting business. One of these factors is the supply chain that is turning into a source of competitive advantage.

Pharmaceutical supply chains consist of special interest to the areas of business, economics and law for two relevant reasons. First, there exist the ordinary issues of structure, conduct and performance. When applied to the pharmaceutical industry, one is supposed to consider high rate of technology modification, critical significance of patent protection, capacity for market power and innovation price and product competitive strategies. Second, the industry is heavily regulated in all major aspects. Much of the published literature concentrates on regulation related to safety and efficacy. However, the supply chain factor is ignored in the former research and the question: “what causes the pharmaceutical supply chain to be a source of competitive advantage” is not still replied (4).

During the last years, however, worldwide pharmaceutical supply chains are facing incrementing and challenging risks. Arguably, the diversity in the pharmaceutical supply-chain risks, in addition pressure from regulatory bodies, changing legislation, customers, and intensive competition are imposing pharmaceutical organizations to carry out supply-chain risk management. Some of the benefits linked with supply-chain risk management are attaining sustainable competitive advantage (5), more efficient decision making, achieving an enhanced balance between opportunity and threat, promoted competitive position, and managing providers more effectively (1). Nevertheless, it has been accepted that the most challenging perspective of supply-chain risk management is the detection of risk factors for reduction, as result, supply risk management (SRM) and supplier selection (SS) become necessary parts of supplier management (6). Therefore, to guarantee pharmaceutical supply-chain flexibility and continuity, it is recommended to effectively evaluate risks and develop a comprehensive mitigation approach (7). 

Finally, based on aforementioned problem, our research question drafted as following:

“Which risk factors in supplier selection should be considered by pharmaceutical companies?”

To answer the question, this article benefits from the fuzzy TOPSIS to quantify risk factors. The remainder of the paper is organized as follows: section 2 provides a review of Iranian pharmaceutical background. Section 3 the literature on SCM and its components in pharmaceutical industry. In Section 4 study design and basic factors are developed. Section 5 presents the results and survey analysis and finally in section 6 conclusion and implications are provided.

*Iranian pharmaceutical background*

Medicine and pharmacy are among the oldest sciences and disciplines in Iranian civilization. After Islam was introduced to Iran, it had a great impact on both sciences. The influence was so great that it drew a line in the history of pharmaceutics in Iran. There are two different but continuous eras of medicine and pharmacy of Iran; before Islam and after Islam. The sciences of medicine and pharmacy were greatly improved during the reign of Islamic civilization. The Islamic pharmacists and physicians followed methods of Hippocrates and Galen. Among the most famous Persian physicians and chemists are Mohammad-ebn-e Zakaria Razi and Avicenna who both were living during Medieval era. The most popular book of Avicenna in medicine is “Ghanoon” written in five volumes. Two volumes of the book are devoted to pharmacology (8). 

*Pharmaceutical companies in iran*

On the eve of the 1979 revolution, numerous domestic, foreign, and domestic-foreign private companies were active in Iran›s pharmaceutical sector. By that time, the country›s pharmaceutical sector had been transformed into a market that boasted a $300 million annual cash flow. There were nearly 4,000 kinds of pharmaceutical products available in Iran, 70% of which was provided by imports and the remaining 30% was produced domestically. More than half of the latter market served the sales of products under the concession of foreign companies (10). At present more than 95% of the drug consumption is produced by domestic pharmaceutical companies (10). 

*Generic system in iran*

**Figure 1:**
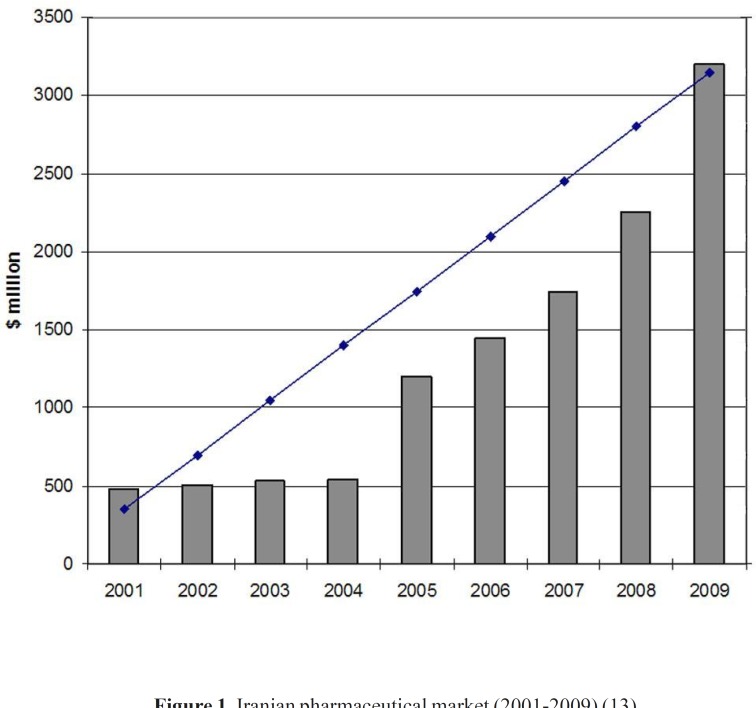
Iranian pharmaceutical market (2001-2009) (13).

The year 1981 witnessed the beginning of a roundup of actions aimed at adopting and implementing policies to modernize the Iranian pharmaceutical sector, which influenced this industry all the way up to 1994. These programs, entitled Generic Scheme, sometimes also called the Generic Concept, formed the foundation of the new pharmaceutical system in the country. In recent years, national pharmaceutical system was directed to the brand-generic and brand systems and, as a result, there is some competition in the industry. This provides good opportunities for future development of domestic pharmaceutical industry. The fact is that the domestic industry has not yet adequately developed to its full capacity and there are much potential capabilities for further growth and development (11). Domestic pharmaceutical industry is experiencing a substantial double digit growth in the recent years. Furthermore, in house production of hi-tech biological products is an emerging know- how in Iran’s pharmaceutical sector. In recent years some private firms have focused to produce biological pharmaceuticals, using novel biotechnology methods (12). The annual growth of Iranian pharma market value (2001-2009) is shown in Figure 1. The share of domestic pharmaceutical sale to total pharmaceutical sale in the year 2009 was around 60 percent (13).

*Literature review*

The pharmaceutical market is intensely regulated in many countries because of the unique nature of demand and supply for drugs (14). According to the characteristic of the competition in drug market, governments are expected create balance in both clinical and economic interests (15). The pharmaceutical sector has an important role in the medical and health system. Increasing size and aging of population, rapidly growing economy and exceeding prevalence of chronic illnesses (such as cardiovascular disease, cancer, and chronic respiratory disease) in the world, the pharmaceutical industry has developed so fast. The development of the pharmaceutical industry has ensured that the immense majority of Iranian people can easily access essential medicines now (10). Nonetheless, the adequate and appropriate supply does not necessarily signify affordable medicines.

There are a couple of key players in the pharmaceutical industry, including (16):

(i) The large, research and development-based multinationals with a universal participation in branded products, both ethical/prescription and over-the-counter. They tend to have manufacturing sites in many locations. (ii) The huge generic manufacturers, which produce out-of-patent ethical commodities and over-the-counter products. (iii) Local manufacturing companies which are active in their home country, manufacturing both generic products and branded ones under license or contract. (v) Drug discovery and biotechnology companies, often relatively new emergent institutes with no remarkable manufacturing capability. 

The major scopes of production and distribution where pharmaceutical companies need to focus their attempts can be summarized as follows (17): Strategic sourcing and demand synchronization; Scientific production ; Novel product and process development; Extending access to the customer. 

*Components of the pharmaceutical supply chain*

**Figure 2:**
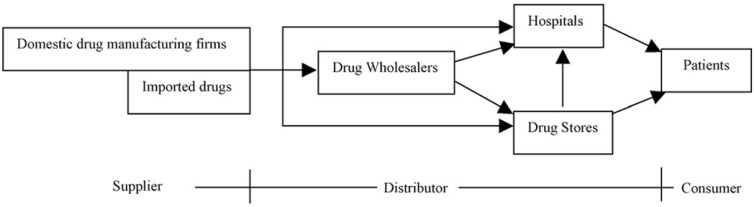
Pharmaceutical supply chain components (15).

As observed in Figure 2, a typical pharmaceutical supply chain will be composed of one or more of the following sectors (15): (i) Primary manufacturing (possibly including contractor sites); (ii) Secondary manufacturing (possibly including contractor sites); (iii) Market warehouses/distribution centers; (iv) Wholesalers and (v) Retailers/hospitals.

The primary manufacturing site undertakes the duty to produce the active ingredients (AI or API). This usually involves either several chemical synthesis and separation steps to construct the complicated molecules, or fermentation and product recovery and purification in the case of biochemical processes. The secondary manufacturing is associated with receiving the active ingredient manufactured at the primary site and introducing “excipient” inert materials accompanied with further processing and packaging to produce the final products. Wholesalers play an essential role in this sector (16).

In order to put this paper in the right context, it is imperative to depict the life-cycle of a medicine; it is to some extent different from that of other process industry commodities. The research or discovery phase tends to utilize thousands of more or less random test compounds against therapeutic targets. It normally lasts about 10 years to result in a potential new medicine that is registered. The potential new drug must then be verified for both safety and efficacy. This involves a variety of trials; at initial stages for toxicity and later on for capability of alleviating symptoms and cure the disease. Finally, the process development trend continues with a chemical or biochemical procedure to manufacture and an associated manufacturing process. This set of activities normally lasts 6–8 years and is usually referred as the development activity. Ultimately, the more familiar processes of production and distribution come up (18).

*Risk management in pharmaceutical supply chain*

*Supply risk management*

Buehler and Pritsch (2003) demonstrate that risk assumption is ultimately a reality of business and management life. Thus, the ability to suppose and manage risks is what organizations need to generate profits and shareholder value (19).

The subject of risk in supply chain is becoming more and more considerable for the following reasons (20): uncertainty in supply and demand (21); globalization of the market; shorter and shorter product and technology life cycles and increased use of outsourcing.

Between supply chain components, supply risk management has attracted a lot of attention to itself and becomes as a more critical part of SCM, because the increasing dependence on suppliers makes companies highly exposed to supply risks. 

In one classification, four key approaches to reduce the effect of SCM risks were suggested (21). I) Demand management: coordination with downstream partners to influence demand in a beneficial manner; II) product management: change in product or process design in order to make more fluent the material flows in the supply chain; III) information management: coordination and collaboration among supply chain partners by sharing information; IV) supply management: collaboration with upstream partners to ensure efficient and effective supply of materials. 

Breen (2008) claims that at every basic level, risks in the pharmaceutical supply chain are connected with product discontinuity, product shortages, poor performance, patient safety/dispensing errors, and technological errors (22). Lack of appropriate risk reduction can destroy public health confidence and reputation, patients’ health and safety, and lead to a decline in profit margin and shareholder value. Although the pharmaceutical firms cannot entirely get rid of the risk portfolio they encounter in their daily operations, they are just able to create an efficient environment for responsive risk mitigation. 

*Supplier selection*

Supplier selection (SS) has been studied for at least 30 years (23). SS also has been investigated through many theoretical and extensive empirical researches, and it is widely accepted as one of the most important activities of the purchasing department in a company (24). SS literature include both perspective research, which propose models to establish how suppliers should be selected and ones, reviewing models that are in use (25). Early SS work focused on criteria that might be used to select suppliers in different purchase situations (26). Several criteria and groupings have been identified and the importance of every criterion generally depends on the type of good/service to be procured (23).

The authors could find no studies on the development of supplier selection risk factors for PSC in any of the developing countries. This research will contribute to reduce the current lack of supplier selection risks studies and also it extends supplier selection scale as a critical component of PSC into developing countries and into a new sector.

*Fuzzy TOPSIS (FT)*

TOPSIS method of solving the multi-criteria decision choosing tasks that implies full and complete information on criteria, expressed in numerical form. The method is very useful for solving of real problems; it provides us with the optimal solution or the alternative›s ranking. In addition to this, it is not so complicated for the managers as some other methods which demand additional knowledge. TOPSIS method would search among the given alternatives and find the one that would be closest to the ideal solution but farthest from the anti-ideal solution at the same time. Modification of the method aims to set a different manner of determining the ideal and anti-ideal point – through standardization of linguistic attributes› quantification and introduction of fuzzy numbers in description of the attributes for the criteria expresses by linguistic variables (27).

*Study design*

In this section we provide a methodology for operationalzing variables and actors, acquiring the data and determining the reliability of factor grouping. The data used in this study gathered from questionnaire distributed to managers in the Iranian pharmaceutical companies. The pharmaceutical industry is chosen because it has a heavy and complete supply chain. These types of firms have tried to improve their supply chain performance due to increasing concerns and importance of supply issues and also manufacturers are seeking methods to improve their performance. 

The questionnaire is designed based on the nine critical factors and 37 questions measuring attitudes listed in Table 1. Basic factors adopted from previous studies which conducted by Micheli *et al. *(2008), Manuj and Mentzer (2008) and Ding *et al. *(2005) and also the chosen response can be strongly disagree, disagree, no opinion, agree, or strongly agree (25, 28 and 26).

In addition to the above questions, information related to the basic profile of the participants was requested at the end of the questionnaire. The main sampling targets were senior managers, department managers and personnel who were involved in company decision making. 

**Table 1:** Factors and related questions

**Factor dimension**	**Questions**
**1. Quality**	• Quality management system • Partnership • Raw material quality • Certificate of GMP
**2. Delivery**	• Transportation quality • Delivery reliability • Timely delivery
**3. Technology**	• Technology development for supplier • Production cost • Technology level
**4. Reputation**	• Agreements • Environmental assessment • Financial risks • Skill workers • Good will
**5. Environmental affairs**	• waste management for suppliers • Environmental regulatory
**6. Flexibility**	• Flexibility in product variety • Flexible quantities • Flexibility in delivering • Customization
**7. Information systems**	• Maturity level • Closed communication • Communication consistency • Mutual trust
**8. Costs**	• Transportation cost • Surplus cost • Cost of goods
**9. Environmental Risks**	• Currency rate • Sanction • Tariff policies changes • Interest rate • Political factors • War and terrorism • Tax payable change • Natural crisis • Consumers taste

*Reliability and validity of the questionnaire*

The internal consistency of a set of measurement items refers to the degree to which items in the set are homogeneous. Internal consistency can be estimated using a reliability coefficient such as cronbach’s alpha (29). In this research it was calculated around 0.8.

The validity of a measure refers to the extent to which it measures what is intended to be measured. Content validity is not evaluated numerically, it is subjectively judged by the researchers (30). It can be argued that because the measurement items were based on an extensive review of the literature on various SCM approaches. To gauge the acceptance of the questionnaire, 10 people who qualified in field of SCM, participated in a pilot test. The participants suggested adding and omitting some parts of questionnaire. Finally, all the pretest participants expressed strong agreement with the suitability of the questionnaire. The questionnaire was considered finalized after modifying the some questions, then ready to be delivered. 

*Data collection*

Data for this study has been gathered using questionnaire that was distributed to 21 pharmaceutical firms which affiliated to tree large holding companies. In order to understand the viewpoints on supplier selection from key sectors of the pharmaceutical industry, questionnaires were sent to the marketing, sales, information, finance, research and development and quality assurance and control departments. Accordingly, we choose respondents from managers who had acceptable knowledge about company’s process, products and general pharmaceutical related issues. The number of questionnaires sent out was 130; the number returned was 73, a return rate of 56 percent. Two of the returned questionnaires were incomplete and thus discarded, making the number of valid questionnaires returned 71 or 54 percent of the total sent out. Finally, 40% of respondent were top managers and 60% were middle managers.

*Data analysis*

Data analysis has been done by statistical analysis and also a Multiple Attribute Decision Making (MADM) model. In statistical analysis we have used t- student tests (one sample t- test), Pearson correlation, and for MADM algorithm we applied fuzzy TOPSIS model. In this section we also used TOPSIS technique to prioritize SCM risk factors. TOPSIS technique needs some criteria and their weights of green productivity criteria.

General TOPSIS process with six activities is listed below (27):

Step 1: Establish a decision matrix for the ranking. The structure of the matrix can be expressed as follows: 

***F***_1_ ***F***_2_ ***… F***_n_


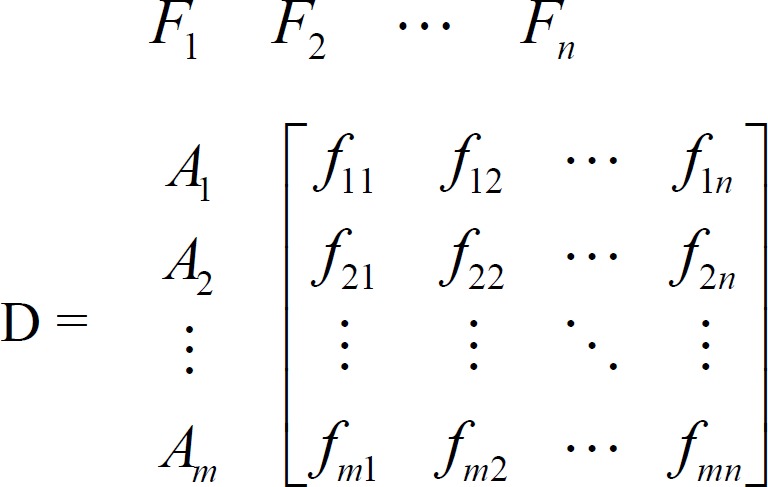


where A_i_ denotes the alternatives i, i = 1, . . . ,m; F_j_ represents j^th^ attribute or criterion, j = 1, . . . , n, related to i^th^ alternative; and ƒ_ij_ is a crisp value indicating the performance rating of each alternative A_i_ with respect to each criterion F_j_. 

Step 2: Calculate the normalized decision matrix R (= [r_ij_]). The normalized value r_ij_ is calculated as:


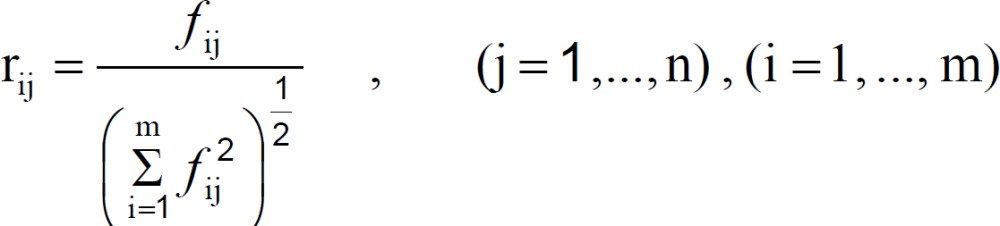


Step 3: Calculate the weighted normalized decision matrix by multiplying the normalized decision matrix by its associated weights. The weighted normalized value V_ij_ is calculated as: 

V_ij_ = r_ij _× W _ij_

Where w_j_ represents the weight of the j^th^ attribute or criterion.

Step 4: Determine the PIS and NIS, respectively:


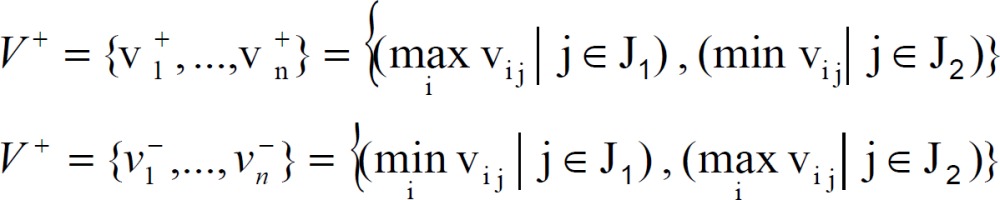


Where J_1_ is associated with the positive criteria and J_2_ is associated with the Negative criteria.

 Step 5 : Calculate the separation measures, using the m-dimensional Euclidean distance. The separation measure D_i_^+^ of each alternative from the PIS is given as: 


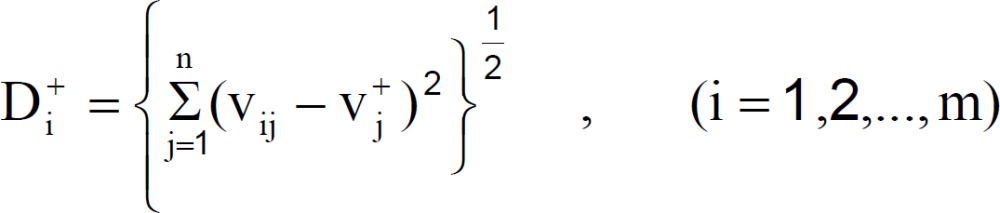


Similarly, the separation measure D_i_^- ^ of each alternative from the NIS is as follows: 


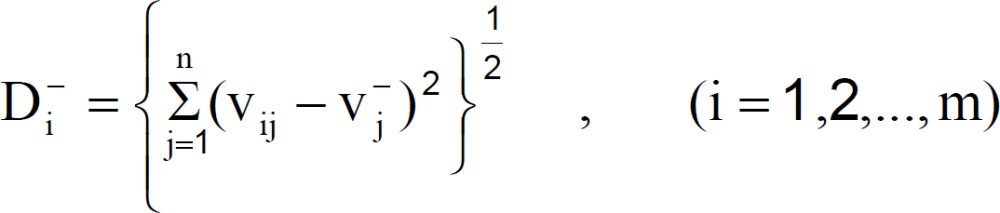


 Step 6 : Calculate the relative closeness to the idea solution and rank the alternatives in descending order. The relative closeness of the alternative Ai with respect to PIS V^+^ can be expressed as:


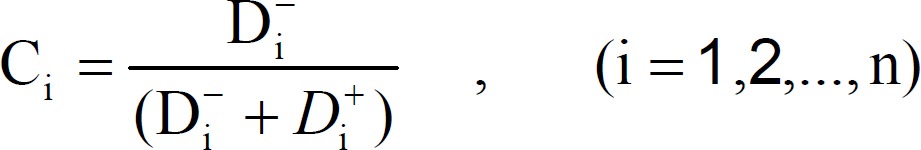


In this study because it was difficult for respondents to answer by crisp model we used fuzzy methods for overcoming the shortage of TOPSIS with crisp approach. 

## Results

*T-test analysis*

In the first step we have done t-test analysis for determining the situations of factors. Table 2 shows the result of t-test and all factors have the significant difference with cut point 3.

**Table 2:** Result of mean difference (one sample t- test).

**Significant level**	**t-statistic**	**Factors**
^*^0.000	21.292	Quality
^*^0.000	7.371	Environmental affairs
^*^0.000	14.956	Flexibility
^*^0.000	28.654	Delivery
^*^0.000	15.838	Technology
^*^0.000	16.229	Information systems
^*^0.000	21.383	Costs
^*^0.000	9.166	Reputation

Significant at ^*^0.05

*Correlation analysis*

We have used Pearson correlation to test the relations among risk factors. It means what’s the inter correlation among basic factors risk. The results indicated that the risk factors have generally correlated together. 

*Result of fuzzy TOPSIS*

In order applying fuzzy TOPSIS, We have converted the language terms to fuzzy numbers according Table 3.

**Table 3:** Language term

(0,0.1,0.2)	1	Very low
(0.1,0.25,0.4)	2	Low
(0.3,0.5,0.7)	3	Medium
(0.6,0.75,0.9)	4	High
(0.8,0.9,1)	5	Very high

The priorities of basic factors according to fuzzy TOPSIS’s results show that the delivering has first priority and cost, quality, Information communication technology (ICT), flexibility, background, technology and finally environmental factors are considered.

In order to ranking the sub factors, we also have applied fuzzy TOPSIS as shown from Table 4 to Table 12.

**Table 4:** TOPSIS rank of quality

**Ci **(rank of TOPSIS)	**Quality**
0.339	Quality management system
0.334	Partnership
0.248	Supplier system quality
0.207	Raw material quality
0.185	Certificate of GMP

**Table 5:** TOPSIS rank of delivering

**Ci **(rank of TOPSIS)	**Delivery**
0.284	Transportation quality
0.169	Delivery reliability
0.140	Timely delivery

**Table 6:** TOPSIS rank of technology

**Ci **(rank of TOPSIS)	**Technology**
0.533	Technology development forsupplier
0.339	Production cost
0.374	Technology level

**Table 7:** TOPSIS rank of reputation

**Ci **(rank of TOPSIS)	**Reputation**
0.597	Agreements
0.515	Environmental factor control
0.367	Financial risk
0.329	Skill workers
0.227	Good will

**Table 8:** TOPSIS rank of environmental affairs

**Ci **(rank of TOPSIS)	**Environmental affairs**
0.567	Waste management for supplier
0.498	Environmental regulatory

**Table 9:** TOPSIS rank of flexibility

**Ci **(rank of TOPSIS)	**Flexibility**
0.414	Flexibility in product variety
0.391	Flexible quantities
0.362	Flexibility in Delivering
0.306	Customization

**Table 10:** TOPSIS rank of Information systems

**Ci **(rank of TOPSIS)	**Information systems**
0.490	Maturity level
0.452	Closed communication
0.397	Communication consistency
0.237	Mutual trust

**Table 11:** TOPSIS rank of costs

**Ci **(rank of TOPSIS)	**Costs**
0.262	Transportation cost
0.215	Surplus cost
0.158	Cost of goods

**Table 12:** TOPSIS rank of environmental risks

**Ci **(rank of TOPSIS)	**Environmental Risks**
0.680	Consumers taste
0.493	Natural crisis
0.402	Tax payable change
0.385	War and terrorism
0.329	Political factors
0.326	Interest rate
0.254	Tariff policies changes
0.207	Sanction
0.156	Currency rate

## Conclusion

The pharmaceutical supply chain (PSC) used to be seen as a tool to supply products to market in an effective way, where the emphasis was on security of supply. Recent changes in the operational environment indicate that companies are revising the components of their supply chains and identifying ways of extracting additional benefits from them.

Risk is an ever-present moderator of business outcomes in all business contexts, and more so with respect to managing complex global supply-chain relationships. No doubt, the current global economic and financial crises underscores the importance of well-developed and well managed risk procedures and structures in all industries, particularly in the developing countries like Iran. As a result, supply-chain risk management more than ever is receiving increasing attention in both academic and industry because of its importance in gaining strategic competitive advantage. Organizations are recognizing the imperative of risk management in the pharmaceutical supply chain. However, one of the critical challenges for managers is deciding on whar risks should be managed and mitigated. Effective risk management requires the ability of the decision maker to rank and prioritize a portfolio of risk factors involved in the supply chain. Given the multidimensional nature of pharmaceutical supply-chain risk, fuzzy TOPSIS methodology was effectively used to model risks into the decision process that proved benefits for managers. The fuzzy TOPSIS methodology results were valid and insightful.

The aim of this research was to gain a more realistic understanding of the nature and prevalence of supplier risk in the PSC as preliminary research, according to McBeath (2004) “understanding the risks and managing to avert them can prevent unplanned cost and improve total performance (31). 

Results of this study based on the fuzzy TOPSIS as an advanced method to prioritize the basic risk factors of supplier selection, show the delivery factor has first priority and cost, quality, ICT, flexibility, seniority, technology and finally environmental factors take subsequent importance.

Results indicate that managers’ views, consider delivery to be the highest priority, manufacturers who use a direct sales model of product distribution, must be able to rely on timely and secure deliveries. Respondent already have accepted that in PSC, timely reliable deliveries is a critical factor to delivering products for customer satisfaction, furthermore, it has been discussed that investing in developing new products and enhancing customer relationships are considered as the main strategies in the changing pharma market (32). 

According to respondent’s attitude, another concern which has the potential to be a risk factor in supplier selection is suppliers’ quality. Due to heavily regulated nature of PSC, companies must establish relationships with suppliers which are accepted by regulatory body through having up-to-date certification *ie*. Good Manufacturing Practice (GMP) (33). For this reason suppliers usually try to establish quality management systems like ISO, EFQM or TQM in their businesses. Considering the flexibility factor, it is important to mention that the ability to compete sustainably depends entirely on meeting customer demands at all times (32). As a result pharmaceutical companies in developing countries like Iran should select flexible suppliers so that they can retain their market share in addition to providing health goods for society. Another factor is related to health, safety and environment (HSE), which is very important in today industries, but here we have the last priorities regarding to environmental affairs, because the pharmaceutical industry is clean one and the consideration of HSE affairs are so high.

*Implication*

During the recent decades, SCM has become a popular agenda for both the pharmaceutical industry and non-pharmaceutical industries. Those pharmaceutical companies that can successfully minimize and manage the risk and uncertainty inherent in their supply chain value stream will achieve superior competitive over competitors in the marketplace. Globalization, outsourcing, single sourcing, just-in-time supply chain management, lean and agile supply chain have made pharmaceutical supply chain more sensitive to risks. Besides aforementioned risk factors, pharmaceutical supply chain may be exposed to risks such as regulatory compliance, currency rate, inflation rate, interest rate, and tariff and duty rate, political condition and natural disasters. 

The authors believe that the proposed risk factors risks in supply chains can help Pharma managers in developing countries like Iran to implement risk factors in a more efficient and effective manner in their suppliers selections.
